# Clinical performance of a multiparametric MRI-based post concussive syndrome index

**DOI:** 10.3389/fneur.2023.1282833

**Published:** 2023-12-18

**Authors:** Steven P. Meyers, Adnan Hirad, Patricia Gonzalez, Jeffrey J. Bazarian, Mark H. Mirabelli, Katherine H. Rizzone, Heather M. Ma, Peter Rosella, Saara Totterman, Edward Schreyer, Jose G. Tamez-Pena

**Affiliations:** ^1^Department of Imaging Sciences, University of Rochester School of Medicine and Dentistry, Rochester, NY, United States; ^2^Department of Vascular Surgery, University of Rochester School of Medicine and Dentistry, Rochester, NY, United States; ^3^Qmetrics Technologies, Pittsford, NY, United States; ^4^Departments of Emergency Medicine, Neurology, Neurosurgery, University of Rochester School of Medicine and Dentistry, Rochester, NY, United States; ^5^Department of Orthopedics, University of Rochester School of Medicine and Dentistry, Rochester, NY, United States; ^6^Department of Physical Medicine and Rehabilitation, University of Rochester School of Medicine and Dentistry, Rochester, NY, United States; ^7^School of Medicine and Health Sciences, Tecnologico de Monterey, Monterrey, Mexico

**Keywords:** post-concussive syndrome, MTBI, MRI, radiomics, diffusion imaging

## Abstract

**Introduction:**

Diffusion Tensor Imaging (DTI) has revealed measurable changes in the brains of patients with persistent post-concussive syndrome (PCS). Because of inconsistent results in univariate DTI metrics among patients with mild traumatic brain injury (mTBI), there is currently no single objective and reliable MRI index for clinical decision-making in patients with PCS.

**Purpose:**

This study aimed to evaluate the performance of a newly developed PCS Index (PCSI) derived from machine learning of multiparametric magnetic resonance imaging (MRI) data to classify and differentiate subjects with mTBI and PCS history from those without a history of mTBI.

**Materials and methods:**

Data were retrospectively extracted from 139 patients aged between 18 and 60 years with PCS who underwent MRI examinations at 2 weeks to 1-year post-mTBI, as well as from 336 subjects without a history of head trauma. The performance of the PCS Index was assessed by comparing 69 patients with a clinical diagnosis of PCS with 264 control subjects. The PCSI values for patients with PCS were compared based on the mechanism of injury, time interval from injury to MRI examination, sex, history of prior concussion, loss of consciousness, and reported symptoms.

**Results:**

Injured patients had a mean PCSI value of 0.57, compared to the control group, which had a mean PCSI value of 0.12 (*p* = 8.42e-23) with accuracy of 88%, sensitivity of 64%, and specificity of 95%, respectively. No statistically significant differences were found in the PCSI values when comparing the mechanism of injury, sex, or loss of consciousness.

**Conclusion:**

The PCSI for individuals aged between 18 and 60 years was able to accurately identify patients with post-concussive injuries from 2 weeks to 1-year post-mTBI and differentiate them from the controls. The results of this study suggest that multiparametric MRI-based PCSI has great potential as an objective clinical tool to support the diagnosis, treatment, and follow-up care of patients with post-concussive syndrome. Further research is required to investigate the replicability of this method using other types of clinical MRI scanners.

## Introduction

Traumatic brain injuries (TBIs) are evaluated based on clinical symptoms, neurological impairments, and imaging findings (1–5). The majority (80–90%) of TBIs are mild, characterized by Glasgow Coma Scale scores of 13–15 (5–7). Mild TBI (mTBI) and concussion are often used interchangeably, with sports-related concussions being a subtype (6, 8). It is estimated that 1.4–3.8 million concussions occur annually in the US and are caused by sports, recreational activities, falls, assaults, and motor vehicle accidents (1, 2, 4, 5).

Symptoms associated with concussions include headaches, amnesia, dizziness, fatigue, drowsiness, sleep disturbance, irritability, blurred vision, nausea, hypersensitivity to light and noise, emotional lability, anxiety, depression, deficits in attention, concentration, memory, executive function, balance problems, and/or loss of consciousness for less than 30 min (5, 7–14). Loss of consciousness occurs in 10–20% of concussions but is not required for diagnosis (15–17). Most symptoms resolve within 14 days in adults and 4–6 weeks in children (4, 6, 7, 16–18). However, 15–30% of mTBI patients may experience post-concussion symptoms, referred to as persistent post-concussive syndrome (PCS), for several months or longer (4, 6, 7, 17–20). In the Prospective TRACK-TBI study, 33% of patients remained functionally impaired 3 months after injury, and 22.4% were not at full functional status 1 year after injury (4), further confirming that some patients with mTBI may experience long-term disability (7). Schneider et al. reported a poor 1-year cognitive outcome in 13.5% of patients with mTBI (21).

In most post-acute patients with a history of mTBI and PCS, there is no radiological evidence of brain injury using computed tomography (CT) or conventional MRI techniques (4, 5, 7, 18, 22). Recently, an advanced MRI technique, Diffusion Tensor Imaging (DTI), has been used to evaluate mild traumatic injuries in the acute, subacute, and delayed phases (5, 7, 18). However, literature reviews have found variable, inconsistent, or negative findings in the diffusion metrics between patients with PCS and controls (5, 6, 18, 22). Additionally, in patients with PCS or post-injury behavioral changes (18), diffusion imaging abnormalities were inconsistent. The inconsistencies in group differences in the locations of DTI-related white matter abnormalities have been proposed to be related to the heterogeneous nature and symptoms of mTBI, different mechanisms of injury, variable locations and phases of injury, differences in DTI protocols, and/or the limited numbers of control and subject populations (5, 18, 23). Previous studies using DTI to evaluate PCS at the group level have focused on univariate analyses of diffusion metrics such as FA, ADC, MD, RD, and AD (24). Although microstructural changes in DTI metrics have been demonstrated at the group level in patients with PCS, the complexity of advanced DTI post-analysis limits its application of these changes at the individual level in clinical settings for diagnosis, treatment, and follow-up care (18, 25).

Despite advances in measuring changes in the brain related to traumatic brain injury, there is currently no objective and reliable MRI assessment to guide clinical decision-making for individual patients with mTBI and PCS (18, 24, 25). The lack of objective data on mTBI in patients leads to challenges in the diagnosis, prognosis, and treatment of patients with a history of mTBI and PCS, based on subjective symptom reports and clinical examinations (25). To address this issue, machine learning (ML) approaches have been suggested (26–28). ML has also been applied to MRI data from patients with traumatic brain injuries. For example, Mitra et al. used a ML technique to classify patients with a history of mild, moderate, or severe TBI, based on altered structural connectivity patterns within intra-and interhemispheric white matter pathways secondary to trauma (28). Additionally, Goswami et al. reported that for retired football players with a history of multiple concussions, ML of mean and radial diffusion data showed alterations involving the uncinate fasciculus, which is associated with behavioral regulation (29). Vergara et al. reported that data from resting-state fMRI used to assess network connectivity was more accurate than DTI in detecting mild traumatic brain injury at the group level (30). Luo et al. reported that a support vector machine algorithm of multiparametric fMRI data in patients with mild traumatic brain injury could improve the classification performance of mTBI compared to normal controls by using the brain regions associated with emotion and cognition (27). Lui et al. (24) reported that an algorithm developed from multi-feature analysis of data from diffusion-weighted imaging, fMRI, and volumetrics may aid in the classification of patients with mTBI compared to controls (24). Abdelrahman et al. reported that combining multiple DTI metrics improved the accuracy of identifying patients with chronic moderate brain injury, with a mean time since injury of 9 years, compared with controls (31).

ML has also been widely applied to multiparametric clinical MRI data in oncology and other medical conditions (32, 33). The purpose of using ML has been to develop clinical tools that support diagnoses, predict prognoses, and predict responses to treatment for different diseases and medical conditions (33). In our previous work, we developed a PCS index (PCSI) for patients with a history of mTBI, using a feature selection process applied to multiparametric structural and diffusion MRI data. The PCSI combines complex radiomic information from the MP RAGE series, fractional anisotropy (FA), and apparent diffusion coefficient (ADC) series (26). This method enabled the detection of post-concussive imaging changes in all series, even when no apparent findings were evident on clinical MRI exams.

The objective of this study was to further evaluate the performance of a multiparametric MRI-based PCSI to detect post-concussive injury in a real-world clinical environment. A significant limitation of the previous study was the control population, which consisted of a relatively small number of young, healthy control athletes. Here, we expand the application of the PSCI to a significantly larger number of routine clinical patients diagnosed with mTBI and patients referred for neuroimaging without history of prior brain injury to explore the associations of the PCSI with sex, mechanism of injury, elapsed time from injury, prior concussion history and clinical symptoms in patients aged 18–60.

## Materials and methods

### Participants

**Figure 1:**
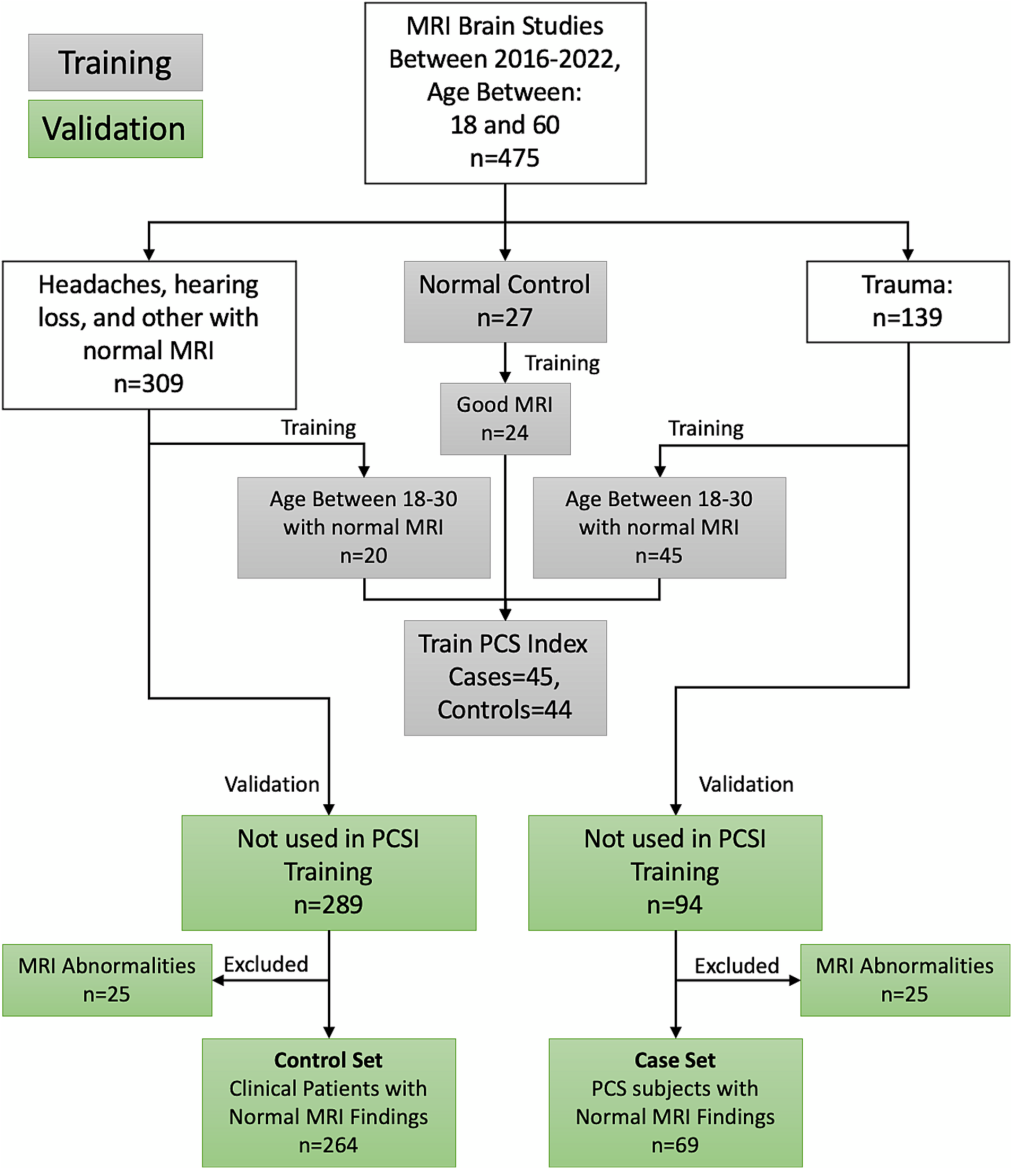
Inclusion–exclusion criteria for the study participants. This paper presents the performance of the PCSI on the validation participants.

The Research Subjects Review Board of the University of Rochester approved this retrospective study. Figure 1 shows the inclusion and exclusion criteria for this retrospective review of the medical records of patients referred to an outpatient MRI center from 2016 to 2022. MRI was performed for 309 uninjured patients ranging in age from 18 to 60 years who were referred for MRI because of non-trauma related subjective complaints of headaches (*n* = 88), hearing loss (*n* = 132), or other complaints (*N* = 42), and who had normal MRI examinations. The uninjured patients also included 27 student athletes recruited as part of the prior study (26). A retrospective review was also performed of the medical records from 2016 to 2022 for 139 patients ranging in age from 18 to 60 years who were referred for MRI at the same facility by Sports Medicine Physicians, Physical Medicine, and Rehabilitation Physicians, or Neurologists with the diagnosis of concussion based on clinical history and combination of symptoms (4, 6–8, 10–18). The inclusion criteria included a clinical diagnosis of concussion with persistent post-concussion symptoms (PCS) and MRI performed at least 2 weeks and no later than 12 months after mTBI, excluding those subjects with dental braces, prior brain surgery, ventricular shunts, intracranial hemorrhage, intra-axial MRI signal abnormalities, skull fractures, or standard contraindications for MR. Thirty eight patients received MR exams within 90 days from injury; 17 between 90 and 180 days from injury, 7 between 180 and 270 days from injury, with the remaining 7 between 270 days and 1 year from injury. Twenty five injured and 25 uninjured subjects were excluded due to issues with the MRI exams, such as missing series required for analysis, or excessive artifact. Patient age and sex, number of previous concussions, absence of loss of consciousness (LOC) at the most recent concussion, the time between injury and MRI, and persistent signs and symptoms were extracted from the electronic medical records. All patient identifiers were removed prior to the analysis.

### Image acquisition

All MRI exams for the concussed patients and 27 student athlete controls were performed between 2016, and 2022, on one of two 3 T Siemens Skyra MRI Scanners using a 20-channel Head/Neck Coil using the following imaging protocol: T1- weighted MP-RAGE images (FOV = 250 mm, 208 axial slices; 1 × 1 × 1 mm, TR = 1,200 ms, TE = 2.29 ms, TI = 600 ms), Flip angle = 8 degrees, 3D axial SWI images [FOV 220 mm, 88 slices/1.5 mm slice thickness (interleaved)/TR 27 ms, TE 29 ms, 1 average], DTI acquisition parameters were axial DTI/TA:10:14 min, FOV 256 mm, 70 slices, 2 mm slice thickness, TR 9,000 ms, TE 88 ms, Flip Angle 15°, 1 average, Acceleration Factor 2/ref. lines 24, diffusion directions 64, *b*-value 1:0 s/mm^2^; b-value 2:1,000 s/mm^2^. Diffusion images were also corrected for susceptibility distortions with the acquisition of a sequence with 64 PA reversed-phase directions. Double IR FLAIR images were obtained for the concussed patients (26).

For the other control patients (*n* = 309), Sagittal T1 FLAIR, Axial MP-RAGE, and DTI images were acquired identically to the concussed patients. All image sets in this study were anonymized and correlated with clinical data. Axial T2-FLAIR images were also obtained for control subjects.

### Image analysis

#### Image preprocessing, segmentation, and quantification

For each subject, three MRI series (MPRAGE, Apparent Diffusion Coefficient, and Fractional Anisotropy) were co-registered using 3D Affine registration with spline deformation and segmented into two tissue types (gray and white matter) in eight anatomical subregions using a derivative of the 152c Montreal Brain atlas. These subregions included the right and left temporal, occipital, parietal, and frontal lobes. Five radiomic quantifications were performed, including raw signal measurement, fractal signature, and three-level wavelet decompositions, which provided information regarding the three-dimensional texture patterns of the measurements. The two tissues, three MRI series, eight subregions, and five radiomic measurements yielded 240 numeric sets, each further described by 33 statistical descriptions ranging from simple arithmetic mean and standard deviation to complex representations of numeric distribution, yielding a total set of 7,920 individual quantitative values for each subject. Logistic Regression with L1 regularization was used to estimate a subset of features to build the Post-Concussive Syndrome Index (PCSI) (34). The PCSI is a mathematical formula that produces a value between 0 and 1 for each subject based on the values of the subset of radiomic measurements, with values less than 0.5 regarded as consistent with uninjured healthy subjects and values of 0.5 or greater associated with post-concussive subjects. Thus, the PCSI value represents the probability of PCS based on the multidimensional MRI signal. Details of the image processing and ML are provided by Tamez-Peña et al. (26). All image processing was performed using CIPAS (Qmetrics Technologies, Rochester, NY), and prediction of the PCSI was carried out using R 4.1.2, with the FRESA.CAD 3.3.1 Package (35).

#### Statistical analysis

For each participant, we recorded the PCSI, sex, age, weight, health, origin of trauma or condition, and clinical symptoms at the time of the MRI. Age, weight, height, and PCSI of subjects in the validation set were described by mean and standard deviation and stratified by cases and controls. Statistical differences between the cases and controls were computed using t-tests for age, height, and weight. The PCSI was described using receiver operating characteristics (ROC) and the area under the ROC curve (ROCAUC). This paper also reports the accuracy, sensitivity, specificity, and diagnostic odds ratio between concussed and non-concussed subjects with their corresponding confidence intervals. The behavior of PCSI values for control group subsets (uninjured subjects with hearing loss, headaches, other indications, or young athletes) was described by frequency and PCSI association using violin plots and compared using ROC analysis. PCSI values of injured subjects were compared according to sex, loss of consciousness at the time of injury, mechanism of injury (sports, vehicular injuries, and assaults/falls), time interval from injury to MRI examination, prior concussion history, and reported symptoms. Significant associations with binary symptoms were computed using the Wilcoxon signed-rank test, and the effect size was described by ROCAUC. None of the *p*-values were adjusted for multiple comparisons. All statistical analyses were performed using R 4.2.2.

## Results

Table 1 presents the demographic characteristics of the study participants. The MRI indication for the control/uninjured participants was hearing loss (*n* = 132), followed by headache (*n* = 88), and several other conditions (*n* = 42). The mechanism of injury in the case/PCS participants was sports-related (*n* = 34), motor vehicle use (*n* = 14), or other causes (*n* = 21). The sex distribution of the case/control sets was statistically different, where female participants were slightly more common in the control group than in the case group, although there were more females than males in both groups. There were marked differences between the age at injury of the participants (cases) and normal clinical patients (controls). Controls were slightly older (42.7 ± 11.2 years) than cases (31.9 ± 14.3 years). Another significant difference in this cohort was the weight of the control participants being heavier than the cases (90.3 ± 25.3 kg vs. 77.3 ± 20.7 kg, *p* < 0.001).

**Table 1:** Demographics of the PCSI validation cohort.

	All (*n* = 333)		Females (*n* = 189)		Males (*n* = 143)	
	Control (*n* = 264)	Case (*n* = 69)				
	(F/M/N) 150/113/1	(F/M/N) 39/30/0				
	MRI indication: Hear loss = 132 Headache = 88 Other = 42	Trauma origin: Sports = 34 MVA = 14 Other = 21	Control (*n* = 150)	Case (*n* = 39)	Control (*n* = 113)	Case (*n* = 30)
Age (years)	42.7 (11.2)	31.9 (14.3)^***^	41.6 (11.0)	33.1 (14.7)^**^	44.4 (11.2)	30.4 (13.8)^***^
Height (cm)	170.1 (10.4)	170.7 (10.9)	163.7 (7.3)	164.6 (7.7)	178.4 (7.6)	178.7 (9.3)
Weight (kg)	90.3 (25.3)	77.3 (20.7)^***^	82.7 (23.7)	71.6 (19.9)^**^	100.7 (23.7)	84.8 (19.6)^**^
PCS index	0.12 (0.17)	0.57 (0.34)^***^	0.13 (0.19)	0.57 (0.37)^***^	0.10 (0.14)	0.60 (0.31)^***^

*p < 0.05, **p < 0.01, ***p < 0.001.

**Figure 2:**
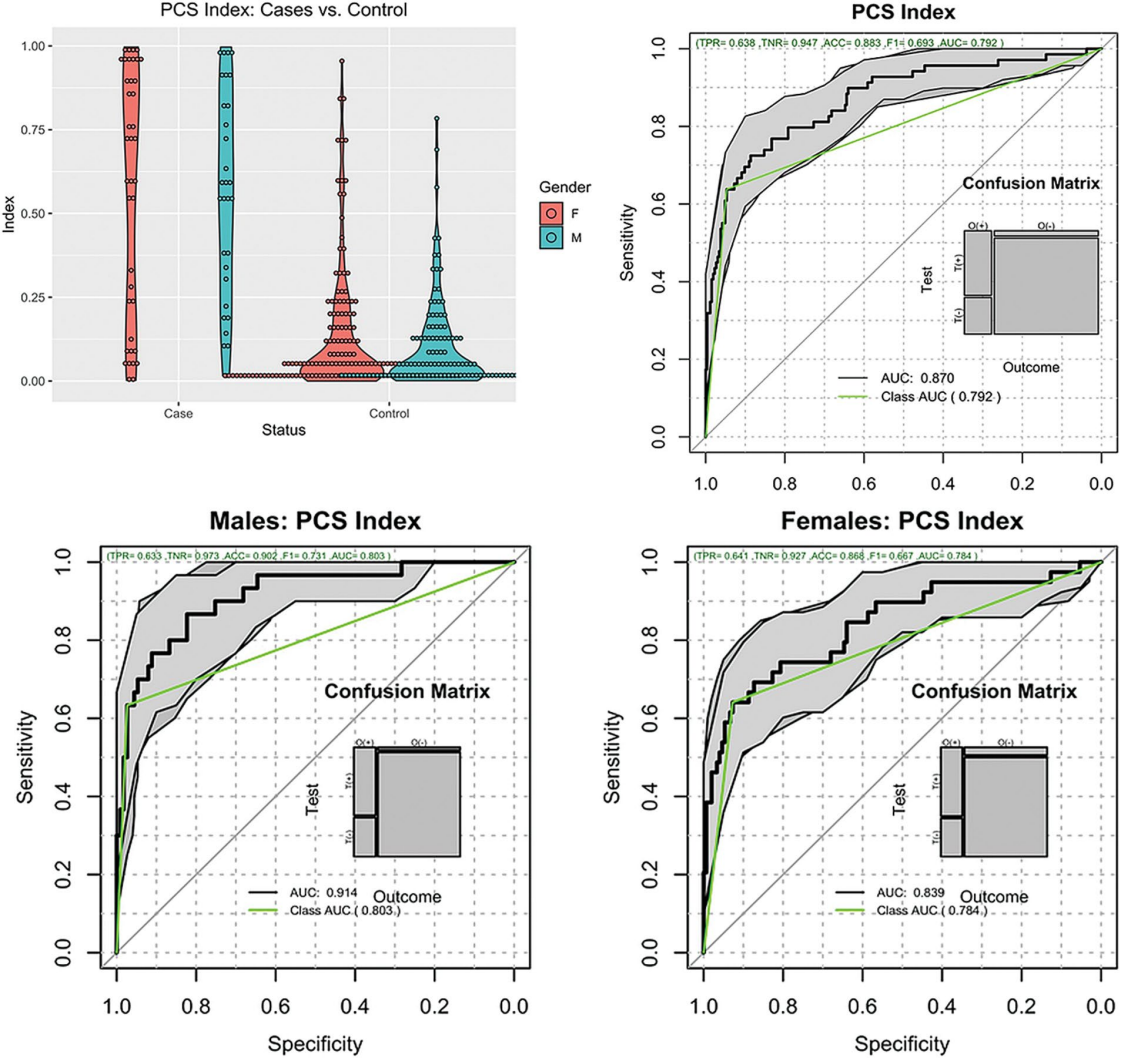
Distribution of the PCSI. Top left, violin plots of the cases and controls stratified by gender. Top right, ROC plot of all subjects. Bottom plots, ROC plots of Males and Females.

Regarding the PCSI, Figure 2 shows the distribution of the index across all patients stratified by sex. The PCSI could separate PCS subjects from non-injury subjects using the injury threshold of 0.5 with an accuracy of 0.88 (95%CI [0.84, 0.92]), sensitivity of 0.64 (95%CI [0.51, 0.75]), specificity of 0.95 (95%CI [0.91, 0.97]), diagnostic odds ratio (DOR) of 31 (95%CI [15, 65]), and ROCAUC 0.87 (95%CI [0.82, 0.92]). The performance of the index was similar between males and females with sensitivity, specificity, and DOR of 0.63, 0.97, and 63 vs. 0.64, 0.93, and 23 for males and females, respectively.

Because there was a strong age imbalance between the cases and controls, we tested the hypothesis that the index was not associated with the participant’s age. The test consisted of modeling the PCSI by participant age adjusted by subject class (Case or Control). The results of the model indicated that subject class predicted most of the variance in the PCSI (*p* < 0.001), while age did not (*p* = 0.42). Hence, the PCSI was not affected by participant age.

### Exploratory analysis of the PCSI

**Figure 3:**
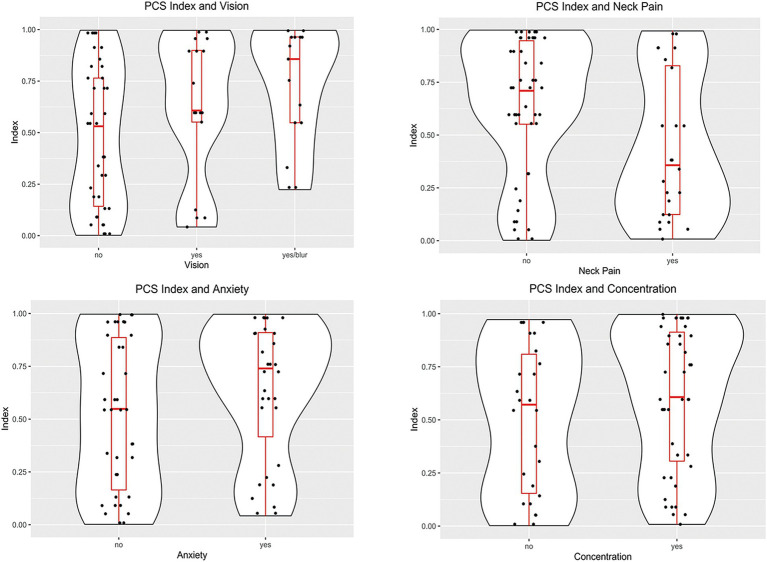
Violin plots of the distributions of the PCSI based on the case symptoms. Subjects reporting vision problems had PCSI values larger than subjects without problems (*p* = 0.02). Subjects reporting neck pain had lower PSCI values than subjects without neck pain (*p* = 0.03). The other symptoms had non-significant differences between the presence or absence of symptoms.

Table 2 shows the distribution of the most common PCS symptoms and their association with the PCSI. The most common symptom of PCS was headache (87%), followed by concentration issues (62%). Headache was not associated with the index value, but the presence of vision problems was associated with a higher index value [0.671 vs. 0.478, p(P > A) = 0.02]. Concentration problems and anxiety showed a trend [(P > A) < 0.1] of higher index values when symptoms were present. Both the symptoms had a ROCAUC of 0.6. Figure 3 shows the distribution of the PCSI for the three top symptoms as well as the distribution of the PCSI and the presence of neck pain. The neck pain results showed a negative trend of having a lower index value for subjects reporting neck pain vs. patients without neck pain [*p* = 0.440, A = 0.636, p(P < A) = 0.03].

**Table 2:** Symptoms and PCSI.

	Symptom	Frequency (%)	Mean index present	Mean index absent	ROCAUC
Cases (*n* = 69)	Headache	60 (87%)	0.567	0.572	0.50
	Concentration	43 (62%)	0.604	0.507	0.59
	Photophobia	39 (57%)	0.535	0.610	0.43
	Fatigue	35 (51%)	0.593	0.542	0.56
	Sleep	35 (51%)	0.584	0.551	0.53
	Dizziness	34 (49%)	0.550	0.585	0.46
	Memory problems	34 (49%)	0.528	0.606	0.44
	Mood problems	33 (48%)	0.572	0.564	0.49
	Noise sensitivity	33 (48%)	0.532	0.600	0.44
	Vision problems	32 (46%)	0.671	0.478	0.66*
	Anxiety	31 (45%)	0.633	0.514	0.60
	Nausea	31 (45%)	0.594	0.546	0.55
	Irritability	28 (41%)	0.543	0.585	0.54
	Neck pain	24 (35%)	0.440	0.636	0.34*
	Balance	21 (30%)	0.489	0.602	0.40
Controls (*n* = 264)	Hearing loss	132 (50%)	0.118	0.100	0.50
	Headache	88 (34%)	0.143	0.094	0.54
	Other	44 (16%)	0.147	0.121	0.44

Bold values indicate ROC AUC greater than 0.6. *p < 0.1.

**Figure 4:**
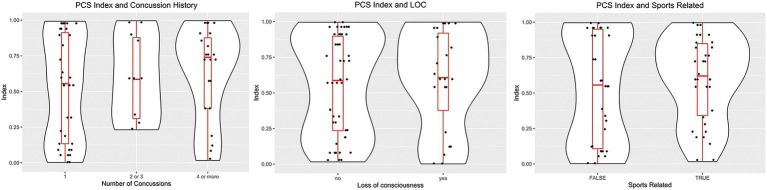
Violin plots showing the distribution of case subjects according to the history/origin of the concussion. Left, distribution based on the number of concussions. Middle, PCSI distribution according to the history of loss of consciousness. Right, differences between the origin of the trauma: Sports injury or another traumatic event.

Figure 4 shows the distribution of the PCSI concerning the history of previous concussions, loss of consciousness, and the mechanism of trauma. In these cases, we observed a positive trend toward a higher PCSI value in the three exploratory analyses, but none of them reached statistical significance (*p* > 0.1).

We also explored the effect of elapsed time from injury on the PCSI and observed a non-significant trend of diminishing index values with increasing time from injury (*p* = 0.12).

The final exploratory analysis explored the behavior of the index in non-trauma participants. The results are presented at the bottom of Table 2. The hearing loss, headache, and other patient subcohorts had non-significantly different PCSI values than the other non-trauma patients [p(A ≠ B) = 0.16, 0.88, and 0.09, respectively].

## Discussion

This study showed that our previously developed ML-based classifier, the PCSI, enabled differentiation of real-world patients with clinically diagnosed mTBI from those without history of head injury with statistical accuracy (26). In this study, we further evaluated the performance of the PCSI by classifying a broader demographic of injured patients with PCS and comparing them with a broader control population of uninjured subjects aged between 18 and 60 years, imaged on multiple MRI scanners. The results of this study indicate that PCSI performed well in the real-world population, with a mean PCSI value of 0.57 for patients with PCS and a mean PCSI value of 0.12 for control subjects (ROCAUC 0.87, 95%CI [0.84, 0.92]). The PCSI had a sensitivity of 64%, specificity of 95%, and accuracy of 88% for the evaluation of patients with PCS. Clear differences in PCSI between controls and injured patients were observed across population subgroups, including females, males, sports-related mTBI, and non-sports-related mTBI, as well as sub-cohorts of injured and non-injured athletes. These results further suggest that the PCSI detects post-injury microtrauma, and that mTBI subjects with PCS exhibit significant differences in the content of the MRI information from combined structural and DTI image data as compared to uninjured subjects.

The PCSI performance of 88% accuracy with 65% sensitivity compares well with the reported work of Fleck et al. (36) that showed classification accuracy of only 62%. The main difference is that our work is based on an open adult population, while their study concentrated on an adolescent population. Other studies on the use of AI in mTBI showed similar performance, with sensitivity varying from 68% (Mitra) (28), 79% (Goswami) (29), to 89% (Vergara) (30). Even though these last three studies used mTBI subjects instead of PCS subjects, they illustrated that AI has an important role in identifying subtle differences in brain function or microstructure that may have clinical relevance in the near future.

Although performance of the PSCI is good, the current method is based on a simple logistic model with L1 penalization. In the future, a more elaborate ML method may be able to be implemented, considering the source of individual misclassification errors, or the inclusion of additional clinically relevant information to further enhance accuracy. Furthermore, the presented results were validated using the same hardware, obtained using the same protocol on the same types of 3.0 Tesla MRI scanners, thus enabling data harmonization. Therefore, the performance of the developed PCSI must be evaluated on other types of MRI scanners, such as 3 or 1.5 Tesla, and with other diffusion tensor imaging protocols.

No significant differences were found in the PCSI based on sex for the control group or the mTBI cohort, consistent with previously reported results regarding the effect of sex on the frequency of PCS and rate of clinical recovery (37). The analysis of the PCSI showed a non-statistically significant, slightly downward trend associated with increased time intervals between injury and MRI. These findings are similar to those of multiple prior studies that showed persistent changes in individual DTI metrics from weeks to months after mTBI although the specific DTI metrics were inconsistent in their behavior (5, 18, 38–41). These observations are consistent with previous findings of partial resolution of alterations in AD and MD in cerebral white tracts from 2 weeks to 6 months post-mTBI, compared with persistent changes in lower FA. Contradictory results have been reported regarding the outcome of patients with PCS in relation to the number of prior concussions (17, 40, 42, 43). In our study, the relationship between the number of prior concussions and the PCSI showed a trend of increasing PCSI values for subjects with a history of more prior concussions; however, this did not reach statistical significance. In our study, no statistically significant differences were found in the PCSI values when comparing sub-cohorts of injured subjects who lost consciousness at the time of injury and those who did not, consistent with previous reports that loss of consciousness is not associated with an increased rate and duration of PCS (17, 44).

The exploratory analysis of the case subjects revealed that certain symptoms were associated with higher PCSI values. Specifically, we found that participants with vision problems had higher PCSI values than those without vision problems. Similar trends were observed for anxiety and concentration. As illustrated in Figure 3, subjects who reported vision problems, including blurring, had higher index values than those who did not (45). Upon examination, all patients with vision problems complained of either binocular blurred vision or convergence insufficiency. At least 12 of the 30 participants with vision problems also reported LOC after a concussive episode. Unfortunately, a complete ophthalmologic report was unavailable for these subjects; therefore, our ability to localize the potential injury site was limited. However, blurred vision is often the result of diplopia or nystagmus, which can occur from dysfunction in pathways located in the midbrain, a region with known biomechanical vulnerability to concussive forces (45). The connection between these symptoms and midbrain dysfunction is more likely for the subset of subjects presenting with both blurred vision and LOC, indicating midbrain-thalami involvement.

It is worth noting that the PCSI was negatively correlated with the presence of neck pain in the subset of cases whose trauma originated from motor vehicle accidents, which suggests the possibility of PCS symptoms originating from neck injury rather than structural brain microtrauma, assuming greater prevalence of neck injury due to MVA than other causes of mTBI (46, 47).

Our prior study suggested that the PCSI was able to detect structural post-injury microtrauma in the brain, but was limited by a small, relatively young control demographic. In this study, we expanded the study of the PCSI to include subjects injured in everyday activities outside of athletics, and uninjured subjects undergoing neuroimaging for tinnitus and migraine, but otherwise healthy, with normal MRI exam findings and no history of head trauma. This larger study population, imaged on multiple MRI scanners, demonstrated that the PCSI continued to accurately differentiate subjects with history of mTBI and PCS from those without, even those with similar symptoms (headache). The results presented continue to suggest the PCSI detects structural microtrauma associated with mTBI. Significant limitations include a lack of objective data confirming neurological injury, such as histopathology or force vector data from the injury event that could be compared with PSCI results to show positive correlation with injury. PCSI values could add confidence to diagnosis of concussion and determination of return to activity based on resolution of symptoms. Although our data trends show the PCSI diminishes with time from injury, we have no proof that the PCSI follows patient recovery from mTBI, given the lack of PCSI information from mTBI patients without PCS, e.g., those who have clinically recovered. Thus, the results presented in this study do not conclusively indicate whether the PCSI can predict the resolution of PCS or whether brain changes observed due to mTBI are permanent fixtures in affected patients. We hope to address this in future work examining longitudinal correlation of PCSI values and symptomatic recovery, perhaps providing prognostic information about recovery timelines. Sub-regional PSCI information could also help physicians determine patient-specific recovery therapy.

## Conclusion

The results of this study show that the previously developed PCSI applied to multiparametric MR data from individuals aged between 18 and 60 years can accurately classify and differentiate patients with PCS from controls from 2 weeks to 1 year after mTBI with high sensitivity, specificity, and accuracy. No statistically significant differences were found in the PCSI values when compared by sex or loss of consciousness at the time of injury and those who did not. The results of this study suggest that the PCSI has great potential as an objective clinical tool to support the diagnosis, treatment, and follow-up care of patients with PCS. Further research is required to investigate the replicability of this method using other types of clinical MRI scanners. The PCSI could also provide sub-regional information about MRI-based structural abnormalities; additional investigation to compare localized PCSI data with objective data on injury localization would further increase confidence in the correlation of the PCSI values with post-traumatic brain injury microtrauma. Finally, work to examine PCSI behavior on patients recovering from mTBI including those who have recovered symptomatically, e.g., without PCS, could provide useful information on the relationship between symptomatic resolution and whether this is due to brain plasticity or the healing of structural injuries.

## Data availability statement

The raw data supporting the conclusions of this article will be made available by the authors, without undue reservation.

## Ethics statement

The studies involving humans were approved by the University of Rochester Office for Human Subject Protection. The studies were conducted in accordance with the local legislation and institutional requirements. The ethics committee/institutional review board waived the requirement of written informed consent for participation from the participants or the participants’ legal guardians/next of kin because This research study involves a retrospective analysis of existing data only and there is no more than minimal risk to subjects. Due to the size of the study it would be impractical to locate and contact the potential subjects and burdensome for them to provide consent. In addition, no personally-identifiable information is being extracted, making it impossible to identify and locate individuals to request consent. Once subjects are selected for inclusion in this study, PHI will be removed for analysis and results reporting. For these reasons, waiving informed consent will not adversely affect the rights of subjects.

## Author contributions

SM: Conceptualization, Data curation, Investigation, Methodology, Writing – original draft, Writing – review & editing. AH: Writing – original draft, Writing – review & editing. PG: Formal analysis, Writing – review & editing. JB: Writing – review & editing. MM: Writing – review & editing. KR: Writing – review & editing. HM: Writing – review & editing. PR: Data curation, Writing – review & editing. ST: Formal analysis, Validation, Writing – original draft, Writing – review & editing. ES: Formal analysis, Project administration, Writing – original draft, Writing – review & editing. JT-P: Conceptualization, Formal analysis, Investigation, Methodology, Software, Supervision, Validation, Writing – original draft, Writing – review & editing.
